# NKILA inhibits NF-κB signaling and suppresses tumor metastasis

**DOI:** 10.18632/aging.101359

**Published:** 2018-01-17

**Authors:** Shun Ke, Rui-chao Li, Fan-kai Meng, Ming-hao Fang

**Affiliations:** 1 Department of Emergency Medicine, Tongji Hospital, Tongji Medical College, Huazhong University of Science & Technology, Wuhan 430030, China; 2 Department of General Medicine, Tongji Hospital, Tongji Medical College, Huazhong University of Science & Technology, Wuhan 430030, China; 3 Department of Hematology, Tongji Hospital, Tongji Medical College, Huazhong University of Science & Technology, Wuhan 430030, China; * Equal contribution

**Keywords:** long non-coding RNA, NKILA, metastasis, esophageal squamous cell carcinoma, NF-κB

## Abstract

The long non-coding RNA (lncRNA) NKILA (nuclear transcription factor NF-κB interacting lncRNA) functions as a suppressor in human breast cancer and tongue cancer. However, the clinical significance and biological roles of NKILA in esophageal squamous cell carcinoma (ESCC) remain unknown. In this study, we showed that NKILA was downregulated in ESCC tissues and cancer cells compared with their normal counterparts. Low NKILA expression correlated with large tumor size and advanced TNM stage, and predicted poor overall and disease-free survival of ESCC patients. Further loss- and gain-of-function assays indicated that NKILA inhibited proliferation and migration of ESCC cells *in vitro*, suppressed tumor growth and lung metastasis *in vivo*. Mechanistically, NKILA could inhibit phosphorylation of IκBα, suppress p65 nuclear translocation and downregulate the expression of NF-κB target genes in ESCC cells. These results suggest NKILA could suppress malignant development of ESCC via abrogation of the NF-κB signaling and may potentially serve as a prognostic marker for ESCC.

## Introduction

Esophageal cancer is considered as a common malignancy as well as the sixth frequent cause of cancer-related mortality worldwide [1,2]. The majority of patients in Eastern countries, especially in China [3-5], was verified pathologically as esophageal squamous cell carcinoma (ESCC), which arises from squamous epithelial cells [5,6]. It is estimated that approximately 500 per 100,000 develop esophageal carcinoma in 2015 and the predicted mortality rate is about 375 per 100,000 in China [3]. However, the overall survival of ESCC remains unsatisfying [2,3]. Therefore, there is an urgent need to identify novel molecular biomarkers and to uncover the underlying mechanisms associated with this fatal disease.

Nuclear factor-κB (NF-κB) is a family of inducible transcription factors involved in inflammation, immunity, cell proliferation and tumor metastasis through binding to the κB sequences located at the promoter regions of more than 200 genes [7,8]. In most resting cells, the NF-κB dimers are sequestered in the cytoplasm by inhibitor of κB proteins (IκB) [9,10]. Activation of NF-κB occurs upon phosphorylation of IκB and following disassembly of the heterodimer [11,12]. Aberrant activation of NF-κB underlies various human disorders including cancer [13], and has become one of the major targets for drug development [14,15]. For instance, siRNA mediated disruption of NF-κB significantly suppressed migration and invasion of ESCC cells though attenuation of the epithelial mesenchymal transition (EMT) [16], the initial step for tumor metastasis [17]. Recently, a long non-coding RNA (lncRNA) NKILA (nuclear transcription factor NF-κB interacting lncRNA) which could mask the phosphorylation site of IκB, inactivate the NF-κB signaling and suppress breast cancer metastasis [18] has attracted our attention. NKILA is a potent predictor for overall survival and a vital determinant of EMT in tongue squamous cell carcinoma [19], melanoma [20] and non-small cell lung cancer [21]. However, the expression level and biological associations of NKILA with clinic pathological parameters in ESCC is unclear.

## RESULTS

### NKILA is significantly downregulated in ESCC tumor tissues and predicts poor prognosis of ESCC patients

**Figure 1:**
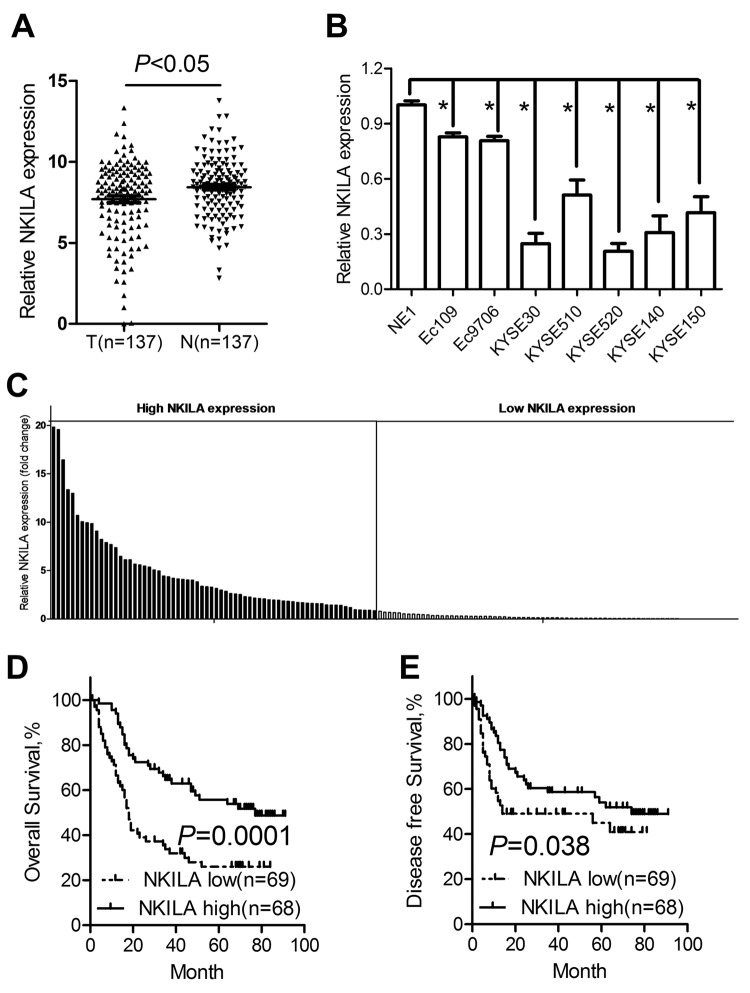
**NKILA is downregulated in ESCC tumor tissues and predicts poor prognosis of ESCC patients.** (**A**) The expression levels of NKILA in ESCC tissues and corresponding noncancerous tissues was detected by qPCR. (**B**) Expression of NKILA in a panel of ESCC cancer cell lines and immortalized squamous epithelial cells. (**C**) The expression levels of NKILA in ESCC cancer tissues was normalized to that of corresponding noncancerous tissues. Data was presented as fold change of △Ct. The patients were assigned to high expression group and low expression group using the median fold change as cutoff value. Kaplan–Meier analysis of overall survival (**D**) and disease-free survival (**E**) in ESCC patients with low and high NKILA levels. Data in B represents the mean ± SD of three repeated experiments. *P < 0.05.

The expression profile of NKILA was detected in 137 paired ESCC cancer tissues and corresponding noncancerous tissues using qPCR assays, while GAPDH was used as the normalization control. As shown in Fig. 1A, NKILA was significantly downregulated in tumor tissues compared with that in normal tissues. Expression level was characterized in a panel of ESCC cancer cell lines including Eca109, Eca9706, KYSE30, KYSE510, KYSE520, KYSE140 and KYSE150 and one immortalized esophageal squamous cell (NE1). The results revealed that NKILA was significantly decreased in all the cancer cells compared with that in NE1 cells (Fig. 1B).

We used fold change of NKILA (tumor/matched normal tissues) as the expression level of each patient and found that 54.01% of ESCC patients showed decreased expression (Fold change < 1) of NKILA (Fig. 1C). Using the median expression level as the cutoff value, patients who express NKILA at levels lower than the cutoff value were assigned into low expression group (n = 69) and those with expression levels higher than cutoff value were designed as high expression group (n = 68) (Fig. 1C). Specifically, we found that decreased NKILA expression was strongly associated with large tumor size (Supplementary Figure S1A) and high TNM stage (Supplementary Figure S1B). However, NKILA expression level was not correlated with age, gender, alcohol consumption, smoking status and differentiation state (Table 1). Correlations between NKILA expression level and survival of ESCC patients were analyzed using Kaplan-Meier methods and log-rank tests. From the Kaplan–Meier survival curve, we observed that patients with low NKILA expression had significantly shorter overall survival (Fig. 1D) as well as disease-free survival (Fig. 1E) than those with high expression. Moreover, uni- and multi-variate analysis indicated that relative NKILA expression level and TNM stage were each determined to be independent prognostic indicators of overall survival in ESCC patients (Table 2).

**Table 1:** The correlation between clinicopathological parameters and NKILA expression.

	NKILA expression		*P*
	Low, n(%)	High, n(%)	
Age			
﹤60	41(59.4)	33(48.5)	0.232
≥60	28(40.6)	35(51.5)	
Gender			
Male	54(78.3)	53(77.9)	1.000
Female	15(21.7)	15(22.1)	
Alcohol consumption			
Ever and current	43(62.3)	47(69.1)	0.473
Never	26(37.7)	21(30.9)	
Smoking status			
Ever and current	28(40.6)	32(47.1)	0.493
Never	41(59.4)	36(52.9)	
Tumor size			
﹤5cm	52(75.4)	62(91.2)	0.021*
≥5cm	17(24.6)	6(8.80)	
Differentiation status			
Well or Moderate	52(75.4)	54(79.4)	0.684
Poor	17(24.6)	14(20.6)	
TNM stage			0.000*
I-II	23(33.3)	45(66.2)	
III	46(66.7)	46(33.8)	

*P<0.05

**Table 2:** Univariate and multivariate analyses of various potential prognostic factors in ESCC patients.

	Univariate analysis			Multivariate analysis	
	HR (95% CI)		*P*	HR (95% CI)	*P*
Age (<60/≥60)	1.36(0.87-2.12)		0.176	-	-
Gender (male/female)	0.96(0.57-1.63)		0.883	-	-
Alcohol (Ever/never)		0.98(0.61-1.56)	0.930	-	-
Smoke (Ever/never)	0.83(0.53-1.31)		0.420	-	-
Tumor size (≥5cm/<5cm)	1.54(0.87-2.71)		0.136	-	-
Differentiation (poor/ well, moderate)	1.36(0.82-2.24)		0.231	-	-
TNM Stage(I-II/III)	3.43(2.12-5.59)		0.000*	2.87(1.73-4.78)	0.000*
NKILA (high/low)	0.42(0.26-0.66)		0.000*	0.59(0.36-0.95)	0.029*

HR: hazard ratio; CI: confidence interval; *P < 0.05.

### NKILA inhibits proliferation of ESCC cells *in vitro*

**Figure 2:**
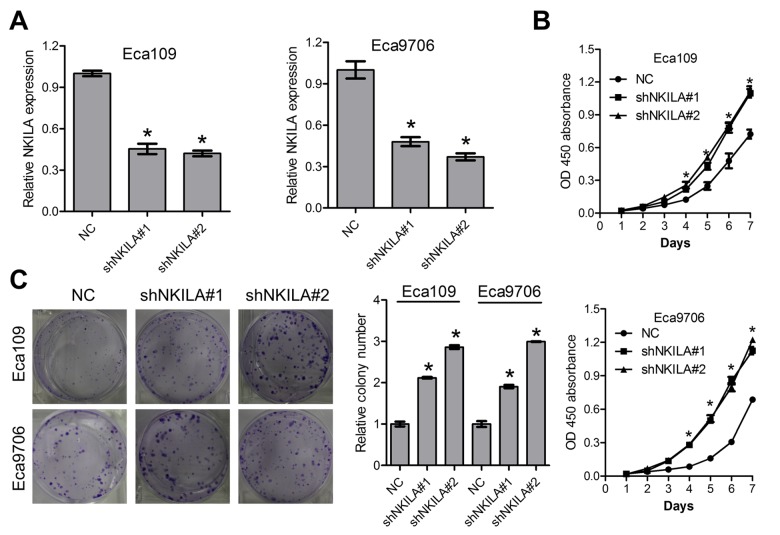
**Knockdown of NKILA promotes proliferation of ESCC cells.** (**A**) The knockdown efficiency of short hairpin RNA against NKILA was examined by qPCR in Eca109 and Eca9706 cells. (**B**) CCK-8 assays of Eca109 and Eca9706 cells after knockdown of NKILA. (**C**) Colony formation assays of Eca109 and Eca9706 cells after knockdown of NKILA. Left panel was representative images and right panel was statistical quantification. Data in **A**, **B** and **C** represents the mean ± SD of three repeated experiments. *P < 0.05.

To further explore the functional relevance of NKILA in ESCC, the Eca109 and Eca9706 cells with relative high NKILA expression were selected for lentivirus transfection and functional analysis. qPCR assays clearly confirmed effective knockdown of NKILA in Eca109 and Eca9706 cells (Fig. 2A). CCK8 assays showed that knockdown of NKILA significantly promoted cell proliferation in Eca109 and Eca9706 cells (Fig. 2B). Silencing of NKILA promoted colony formation capability of Eca109 and Eca9706 cells (Fig. 2C).

### Knockdown of NKILA stimulates tumor growth of ESCC cells *in vivo*

**Figure 3:**
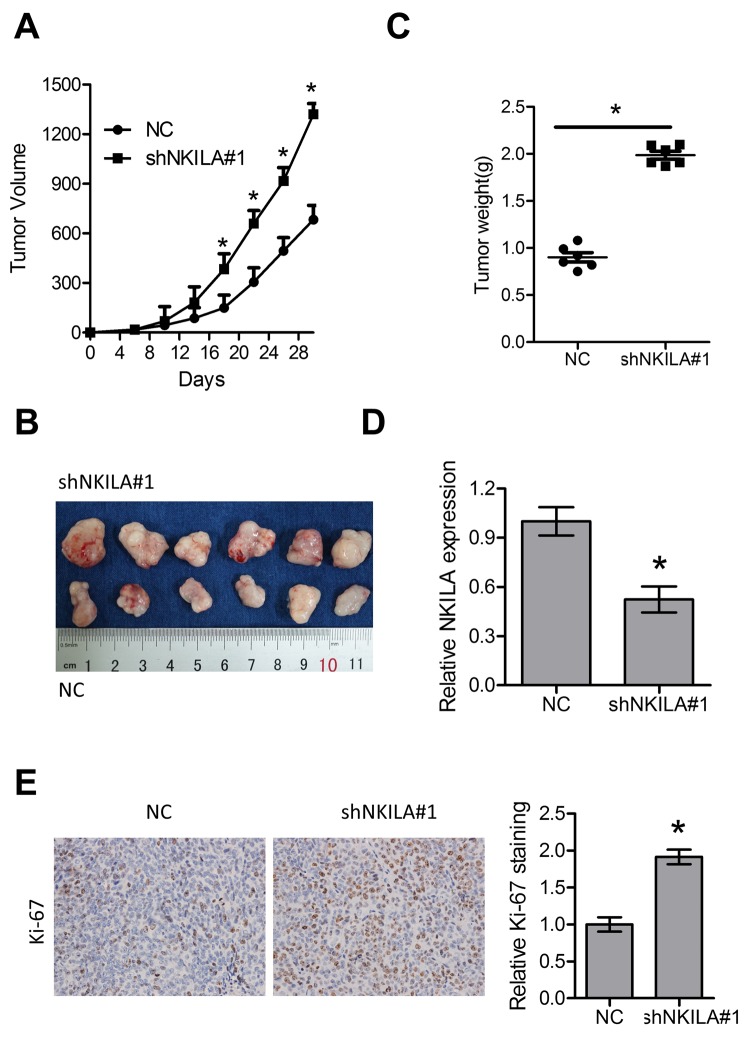
**Knockdown of NKILA promotes tumor growth in nude mice.** (**A**) Tumor growth curve of Eca109 cells after knockdown of NKILA. (**B**) The dissected tumors from the nude mice was photographed. (**C**) Weight of dissected tumors was recorded. (**D**) Expression level of NKILA in the dissected tumors was detected by qPCR. (**E**) Immunohistochemical analysis of Ki-67 in the dissected tumors. Left panel was representative images and right panel was statistical quantification. Data in **A**, **C**, **D** and E represents the mean ± SD of six mice. *P < 0.05.

The tumor suppressive roles of NKILA were further investigated in a xenografted nude mice model. Eca109/NC and Eca109/shNKILA cells were inoculated into the right flank of female nude mice. Compared with Eca109/NC cell-derived tumors, tumors formed by Eca109/shNKILA cells grew more rapidly (Fig. 3A, 3B). Consistently, the mean tumor weight in the control group was also significantly less than that in the knockdown group (Fig. 3C). qPCR assays in the dissected xenografts confirmed efficient downregulation of NKILA in the knockdown group (Fig. 3D). Tumor sections immunohistochemically stained for Ki-67 showed delayed proliferation of tumor cells in the control group, which is in concordance with the *in vitro* results (Fig. 3E).

### NKILA suppresses ESCC metastasis *in vitro* and *in vivo*

**Figure 4:**
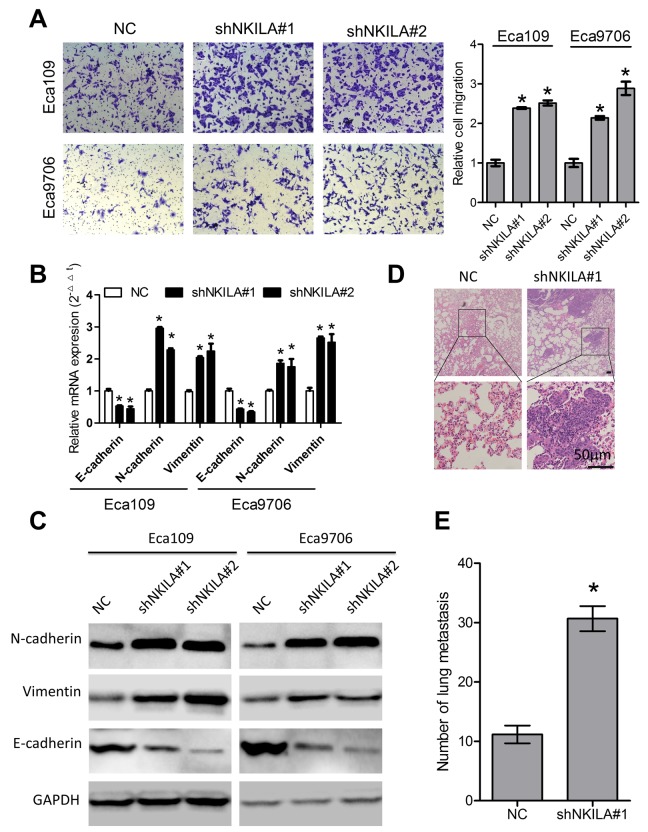
**NKILA suppresses ESCC cell migration *in vitro* and lung metastasis *in vivo*.** (**A**) Silencing NKILA in Eca109 and Eca9706 cells resulted in elevated cell migration. Left panel was representative images and right panel was statistical quantification. (**B**) Expression level of E-cadherin, N-cadherin and Vimentin in Eca109 and Eca9706 cells after knockdown of NKILA was detected by qPCR. (**C**) Expression level of E-cadherin, N-cadherin and Vimentin in Eca109 and Eca9706 cells after knockdown of NKILA was detected by western blot. (**D**) Representative H&E staining of lung tissue sections. Scale bars: 50μm. (**E**) The micro-metastasis in the lung was numbered. Data in **A** and **B** represents the mean ± SD of three repeated experiments. Data in E represents the mean ± SD of six mice. *P < 0.05.

We then performed transwell assays to explore the roles of NKILA in migration of ESCC cells. As shown in Fig. 4A, knockdown of NKILA induced remarkable increase of migration capacity of Eca109 and Eca9706 cells. EMT predisposes cancer metastasis [22]. Our results indicated that knockdown of NKILA led to decreased expression of the epithelial marker E-cadherin and increased expression of the mesenchymal markers including N-cadherin and Vimentin at both the mRNA (Fig. 4B) and protein level (Fig. 4C). Moreover, in the experimental metastasis models, assessment of the micro metastasis by hematoxylin and eosin (H&E) staining of lung tissue sections showed significant elevation of lung metastasis in the knockdown group (Fig. 4D, 4E).

### Enforced NKILA expression inhibits proliferation and metastasis in ESCC cells

**Figure 5:**
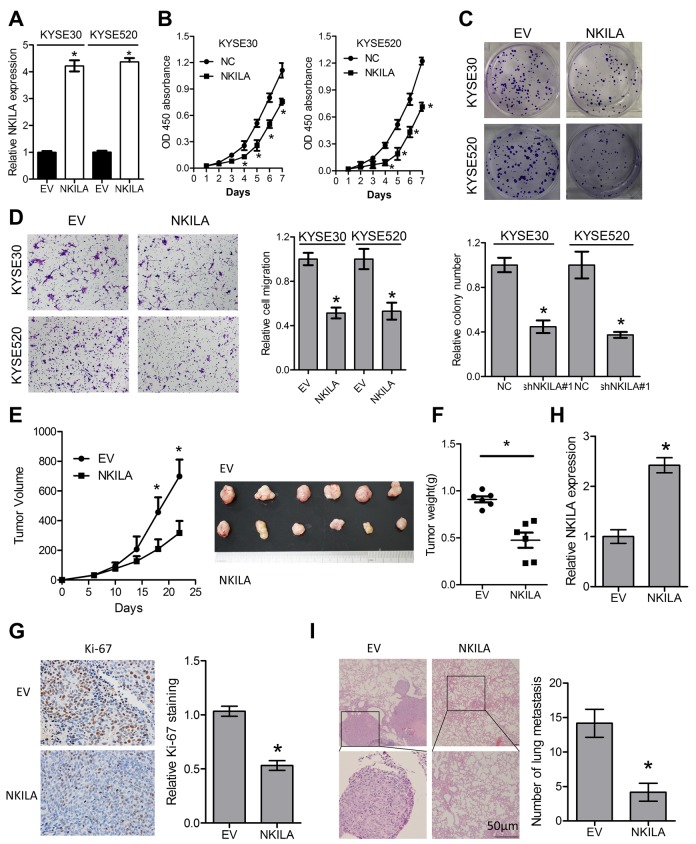
**Enforced NKILA expression inhibits proliferation and metastasis in ESCC cells.** (**A**) Overexpression of NKILA in KYSE30 and KYSE520 cells was confirmed by qPCR assays. (**B**) CCK-8 assays of KYSE30 and KYSE520 cells after overexpression of NKILA. (**C**) Colony formation assays of KYSE30 and KYSE520 cells after overexpression of NKILA. Upper panel was representative images and lower panel was statistical quantification. (**D**) Migration assays of KYSE30 and KYSE520 cells after overexpression of NKILA. Left panel was representative images and right panel was statistical quantification. (**E**) Tumor growth curve of KYSE30/EV and KYSE30/NKILA cells. The dissected tumors from the nude mice was photographed. (**F**) Weight of dissected tumors was recorded. (**G**) Immunohistochemical analysis of Ki-67 in the dissected tumors. Left panel was representative images and right panel was statistical quantification. (**H**) Expression level of NKILA in the dissected tumors was detected by qPCR. (**I**) Representative H&E staining of lung tissue sections formed by KYSE30/EV and KYSE30/NKILA cells. Scale bars: 50μm. The micro-metastasis in the lung was numbered. Data in **A**, **B**, **C** and **D** represents the mean ± SD of three repeated experiments. Data in **E**, **F**, **G**, **H** and I represents the mean ± SD of six mice. *P < 0.05.

To gain further insight into the biological roles of NKILA in ESCC, NKILA lentiviruses were introduced into KYSE30 and KYSE520 cells (Fig. 5A). Overexpression of NKILA significantly suppressed proliferation of KYSE30 and KYSE520 cells as indicated by lower CCK-8 value (Fig. 5B) and decreased colonies (Fig. 5C). Moreover, overexpression of NKILA significantly suppressed migration (Fig. 5D) and EMT (Supplementary Figure S2) in KYSE30 and KYSE520 cells. In the subcutaneous mouse model, enforced NKILA expression significantly suppressed tumor growth (Fig. 5E-G), which was consistent with the *in vitro* results. NKILA was overexpressed in the dissected xenografts from KYSE30/NKILA group than that from KYSE30/EV group (Fig. 5H). After tail vein injection of KYSE30/EV and KYSE30/NKILA cells, the lung metastasis was examined by H&E staining and showed significant decrease in the overexpression group (Fig. 5I).

### NKILA inhibits IκBα phosphorylation and NF-κB activation in ESCC cells

**Figure 6:**
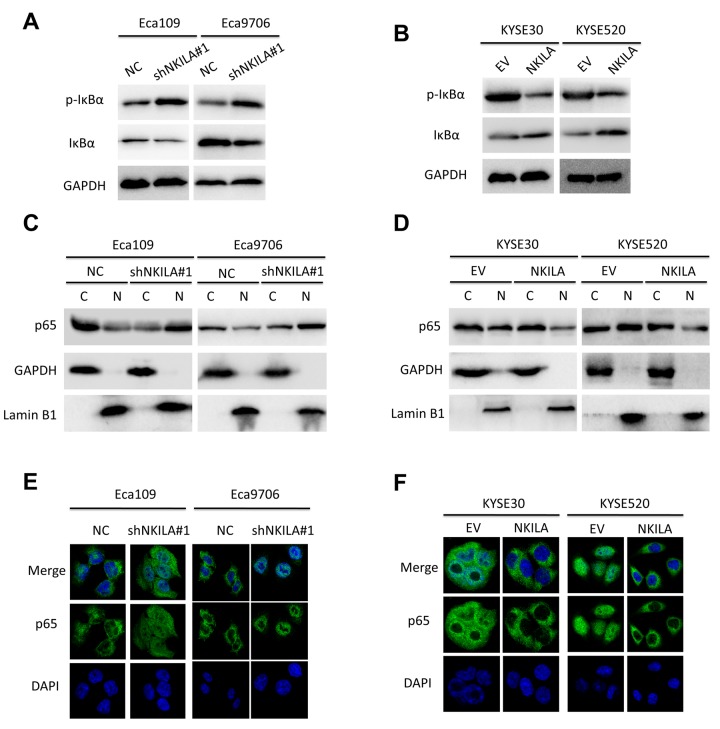
**NKILA inhibits activation of NF-κB signaling of ESCC cell.** (**A**) Immunoblotting of p-IκBα and IκBα in Eca109 and Eca9706 cells after knockdown of NKILA. (**B**) Immunoblotting of p-IκBα and IκBα in KYSE30 and KYSE520 cells after overexpression of NKILA. (**C**) Immunoblotting of p65 in the cytoplasm and nucleus in Eca109 and Eca9706 cells after knockdown of NKILA. (**D**) Immunoblotting of p65 in the cytoplasm and nucleus in KYSE30 and KYSE520 cells after overexpression of NKILA. (**E**, **F**) Immunofluorescence of p65 in ESCC cells after manipulation of NKILA expression. The nucleus was counterstained with DAPI.

**Figure 7:**
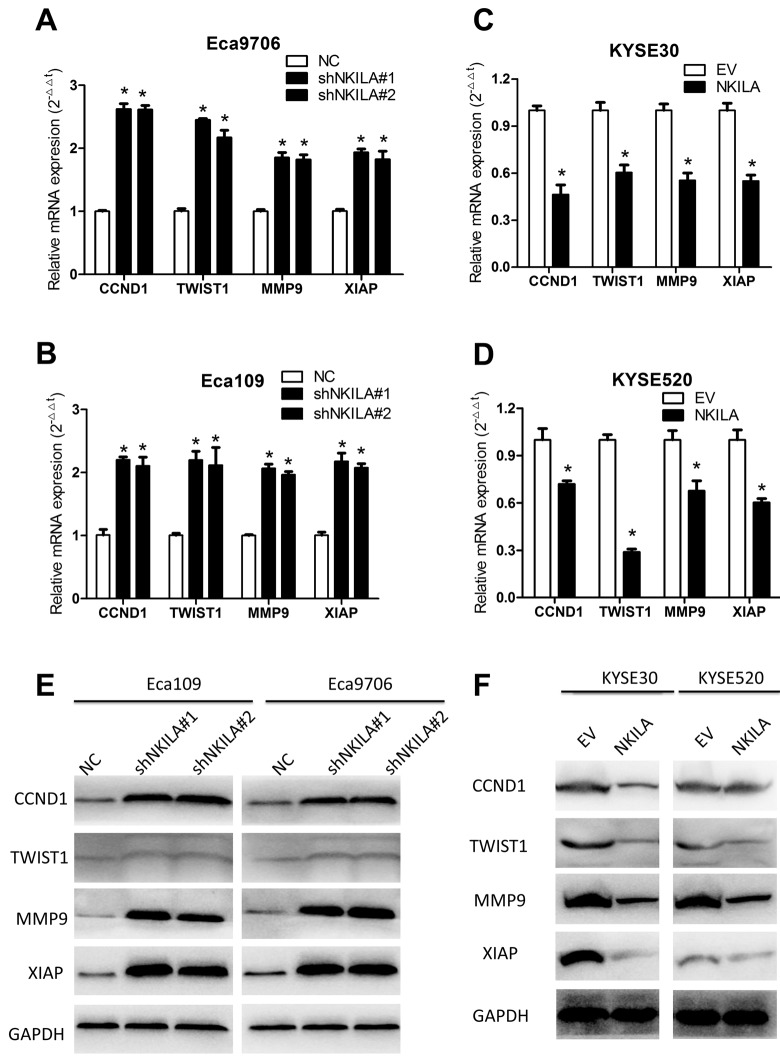
**NKILA suppresses expression of several NF-κB target genes in ESCC cells.** (**A**, **B**) qPCR analysis of NF-κB target genes in Eca109 and Eca9706 cells after knockdown of NKILA. (**C**, **D**) qPCR analysis of NF-κB target genes in KYSE30 and KYSE520 cells after overexpression of NKILA. (**E**) Immunoblotting of CCND1, TWIST1, MMP9, XIAP in Eca109 and Eca9706 cells after knockdown of NKILA. (**F**) Immunoblotting of CCND1, TWIST1, MMP9, XIAP in KYSE30 and KYSE520 cells after overexpression of NKILA. Data in **A**, **B**, **C** and **D** represents the mean ± SD of three repeated experiments. *P < 0.05.

Because over-activated NF-κB signaling have been associated with malignant transformation of several human cancers including ESCC [7] and NKILA was reported to suppress activation of NF-κB in breast [18] and tongue cancer [19], we asked whether NKILA could inhibit NF-κB in ESCC. Protein level of IκBα and NKILA expression level correlated inversely in ESCC tumor tissues (Supplementary Figure S3A, S3B). Phospholevel of IκBα was enhanced in Eca109 and Eca9706 cell after knockdown of NKILA (Fig. 6A) and decreased in KYSE30 and KYSE520 cells after overexpression of NKILA (Fig. 6B), while phosphorylation of IκBα kinase (IKK) was not affected by NKILA expression (Supplementary Figure S3C). Phosphorylation of IκBα resulted in decrease of total protein in ESCC cancer cells (Fig. 6A, 6B). The cytoplasm and nuclear proteins were fractioned to explore translocation of p65 after NKILA manipulation. As shown in Fig. 6C and 6D, knockdown of NKILA induced elevated nuclear p65 expression in Eca109 and Eca9706 cells, while enforced NKILA expression suppressed p65 nuclear translocation in KYSE30 and KYSE520 cells. Suppression of p65 translocation by NKILA was further confirmed by immunofluorescence in ESCC cells (Fig. 6E, 6F). These results suggested that suppression of NF-κB by NKILA was mainly associated with decreased phosphorylation of IκBα, which is in concordance with previous reports [18,19]. On the other hand, both qPCR (Fig. 7A-D) and western blot (Fig. 7E, 7F) assays confirmed that NKILA suppressed activation of several NF-κB target genes including CCND1, TWIST1, MMP9 and XIAP, all of which play vital roles in cancer growth and metastasis.

To explore the effects of NF-κB in mediating NKILA regulated migration in ESCC, cells were treated with BAY 11-7082 (a NF-κB inhibitor) or TNFα (a NF-κB activator). BAY 11-7082 reversed pro-metastasis effects of NKILA knockdown in Eca109 and Eca9706 cells (Supplementary Figure S4A), while TNFα blocked the anti-metastasis roles of NKILA reintroduction in KYSE30 and KYSE520 cells (Supplementary Figure S4B), further indicating that NKILA regulated migration of ESCC cells via NF-κB pathway.

## DISCUSSION

In this study, we characterized the expression pattern and biological functions of the lncRNA NKILA in ESCC and found that downregulated expression of NKILA may promote malignant phenotype of ESCC. Further loss- and gain-of-function analysis showed that NKILA played a key role in cell proliferation and migration. More importantly, blockade of NF-κB activation by NKILA via inhibition of IκBα phosphorylation was associated with its tumor suppressive roles. Our data indicated that NKILA may be regarded as a clinically valuable prognosis biomarker and a potential therapeutic target in ESCC.

It has become increasingly apparent that mammalian genomes encode numerous lncRNAs, which are more than 200 nucleotides in length with limited protein-coding capacity [23,24]. Roles of lncRNA in human disorders including malignancies are emerging as the hot issues than ever before [25-29]. For instance, lncRNA MALAT1 might be applied as useful biomarkers to predict the prognosis of gastrointestinal malignancies including ESCC [30-32]. Links between lncRNAs including HOTTIP [33], HOTAIR [34], lnc-ATB [35] and BC032469 [36] and ESCC have been established. Specifically, the lncRNA XIST [37] and lnc-ATB [35] have been shown to promote EMT of ESCC cells. In this study, we focused on the newly identified lncRNA NKILA and found that NKILA was significantly downregulated in the ESCC tumor tissues compared with corresponding normal tissues. Loss- and gain-of-function assays showed NKILA could act as a tumor suppressor in ESCC.

Previous reports have thoroughly studied molecular mechanisms underlying dysregulated NKILA expression in breast cancer [18]. NKILA interferes directly with IκBα and blocks the phosphorylation sites, leading to interruption of NF-κB signaling [6]. Our data showed that phosphorylation of IκBα as well as nuclear p65 expression correlated inversely with NKILA expression in ESCC cells, suggesting that NF-κB signaling plays important roles in mediating anti-proliferation and anti-metastasis effects of NKILA in ESCC. Different target genes have been reported to be involved in NKILA regulated EMT. For instance, Huang W et al. reported that NKILA inhibited TWIST1 expression in tongue squamous cell carcinoma [19]. A recent study showed that NKILA was downregulated in non-small cell lung cancer and suppressed EMT through interfering NF-κB/Snail signal pathway [21]. In the present study, both qPCR and western blot analysis showed that the NF-κB target genes including CCND1, TWIST1, MMP9 and XIAP was upregulated following silence of NKILA and downregulated following NKILA overexpression. Both TWIST1 and MMP9 play vital roles in EMT and tumor metastasis. Although downstream effectors were tumor specific, NKILA mediated NF-κB abrogation in suppression of EMT was consistent in tongue squamous cell carcinoma [19], non-small cell lung cancer [21] and ESCC. Moreover, NKILA was found to inhibit ESCC proliferation *in vitro* and *in vivo*. We speculated that the anti-proliferation effects of NKILA may be associated with downregulation of CCND1. Numerous studies have identified CCND1 as the cyclin that could form a complex with and function as a regulatory subunit of CDK4 or CDK6, whose activity is required for cell cycle G1/S transition [38,39]. Downregulation of CCND1 may result in cell cycle arrest and ultimately growth inhibition of ESCC cells. Although we found that NKILA could suppress ESCC growth and downregulate the CCND1 expression, further studies are needed to explore the anti-proliferation effects of NKILA.

Conclusively, our data showed that NKILA is frequently downregulated in ESCC tissues and verified the correlations between NKILA expression and NF-κB signaling. Our functional experiments showed that NKILA could function as a tumor suppressor through inhibition of cell proliferation and migration *in vitro* and *in vivo*. Thus, NKILA exerts clinical significance and may serve as a prognostic marker and therapeutic target of ESCC in the future.

## MATERIALS AND METHODS

### Patient samples

A total of 137 ESCC cancer tissues and pair-matched adjacent epithelial tissues were collected postoperatively from patients at the Tongji Hospital from 2009 to 2011, Wuhan, China. The specimens were snap-frozen at liquid nitrogen and stored at −80°C until use for RNA isolation. All patients provided written consent for use of their tissues with research purpose and our study were approved by the Institutional Review Board of Tongji Medical College of Huazhong University of Science and Technology. None of our cohort received any preoperative treatments. Complete clinicopathological parameters and follow-up data for all patients were recorded. Overall survival and disease-free survival were defined as the time from the date of surgery to the date of death or last contact and disease recurrence, respectively.

### Cell lines and reagents

Human esophageal cancer cell lines (Eca109, Eca9706, KYSE30, KYSE510, KYSE520, KYSE140 and KYSE150) were obtained either from the Cell Bank of the Chinese Academy of Sciences (Shanghai, China) or purchased from the Deutsche Sammlung von Mikroorganismen und Zellkulturen (DSMZ, Braunschweig, Germany) and cultured at 37°C with 5% CO2 in Dulbecco’s Modified Essential Medium (DMEM) medium (Hyclone, Logan, Utah, USA) supplemented with 10% fetal bovine serum (FBS) (Gibico, Carlsbad, California, USA). Human esophageal epithelial squamous cell NE1 was a kind gift from Dr. Yang XZ at Cancer Hospital and Institute of Guangzhou Medical University (Guangzhou, China) were cultured in a 1:1 mixture of defined keratinocyte serum free medium with growth supplements and EpiLife medium with 60 μM Calcium (Invitrogen,Carlsbad, California, USA). BAY 11-7082 and TNFα were purchased from Selleck (Huston, Texas, USA) and dissolved according to the manufacturer instructions. The fluorescent stain 4',6-diamidino-2-phenylindole (DAPI) and second antibodies used for immunofluorescence were purchased from Invitrogen (Carlsbad, California, USA).

### RNA extraction and qPCR

Total RNA were separated from cells, ESCC cancer and normal tissue specimens by Trizol reagent (Life Technologies, Carlsbad, California, USA) according to the manufacturer’s protocol. The complementary DNA (cDNA) was obtained with the PrimeScript RT reagent Kit (TaKaRa, Dalian, China) and subjected to subsequent qPCR analysis. The RNA expression levels were performed in triplicate with the Roche Light Cycler® 480 apparatus. All Primers used for qPCR were synthesized by Life technology and summarized in Supplementary Table S1, while GAPDH was used as an internal control.

### Cell transfection

Lentivirus expressing short hairpin RNAs or NKILA open reading frame were obtained from Gene Pharma (Shanghai, China). Cells were transfected with lentivirus for 48 hours before they were selected with puromycin (3μg/ml) for another 72 hours. Survived cells were collected for validation of transfection efficiency and following assays. For overexpression of NKILA, cells were transfected with DNA plasmids using Lipofectamine 2000 (Invitrogene, Carlsbad, California, USA) according to manufacturer's instructions.

### Cell proliferation and migration assays

Cells were seeded in 96-well plates (500/well) and the cell proliferation assays were evaluated every 24h with the CCK-8 kit (Djingo, Japan) according to the manufacturer’s protocol. Number of viable cells was quantified by the absorbance at 450 nm using a microplate reader. The transwell assays were performed as previously reported [15]. Briefly, cells were collected and resuspended in 200μl serum-free medium. 2×10^5^ or 1×10^5^ cells were seeded into the upper inserts of 24-well Boyden chambers (Corning, New York, USA). Medium containing 20% FBS were added to the lower chamber serving as chemo attractant. After incubation for 36h, migrated cells were fixed with paraformaldehyde and stained with 0.1% crystal violet solution.

### Western blot analysis and subcellular fraction

Proteins were extracted from cell lysis with the Radio Immunoprecipitation Assay (RIPA) buffer (Thermo Scientific, Carlsbad, California, USA) containing a protease inhibitor cocktail (Selleck, Huston, Texas, USA). BCA kit (Thermo Scientific, Carlsbad, California, USA) was used to quantify proteins and equal amounts was resolved by sodium dodecyl sulfate polyacrylamide (SDS-PAGE) gel and transferred to the PVDF membrane (Millipore, Billerica, MA). The membranes were blocked for 2h with 5% skim milk at room temperature and incubated with primary antibodies at 4°C overnight with gentle shake. The PVDF membrane was then incubated with appropriate secondary antibody for 1 h at room temperature. The bands in the membranes were then detected using the enhanced chemo luminescence substrate kit (Thermo Scientific, Carlsbad, California, USA) and scanned with Image J software. The antibodies used in our study including MMP9, CCND1, XIAP, TWIST1, IκBα, p-IκBα, p-IKKα/β, IKKα, IKKβ, N-Cadherin, E-Cadherin, Vimentin, Lamin B1 and GAPDH are purchased from Cell Signaling (CST, Danvers, MA, USA).

Subcellular fractions were prepared from ESCC cells with a Minute™ Cytoplasmic & Nuclear Extraction Kits (Invent biotech, Eden Prairie, USA) according to manufacturer’s instructions.

### Immunofluorescence

The indicated cells were incubated with primary antibodies against p65 (1:300, CST, Danvers, MA, USA), incubated with Alexa Fluor 488 secondary antibodies (1:1000, Life Technologies, Carlsbad, California, USA) and then counterstained with DAPI (Life Technologies, Carlsbad, California, USA). The images were obtained by OLYMPUS FV1000.

### Animal study

All BALB/c nude mice (4 weeks old, female) were maintained under pathogen free conditions and all procedures for the mouse experiments were approved by the Animal Care Committee of Tongji Medical College. All the animal experiments were performed according to the National Institutes of Health animal use guidelines on the use of experimental animals. Eca109/NC and Eca109/shNKILA cells (1×10^6^cells/mouse) or KYSE30/EV and KYSE30/NKILA cells (1×10^6^cells/mouse) were subcutaneously injected into the right flank of BALB/C nude mice (n=6/group). The tumors volumes were monitored twice every week after injection. All mice were sacrificed four weeks afterwards, and the xenografts were dissected out for qPCR or immunohistochemical analysis. For the metastasis model, the Eca109/NC and Eca109/shNKILA cells (2×10^6^cells/mouse) or KYSE30/EV and KYSE30/NKILA cells (2×10^6^cells/mouse) were injected into the tail vein of nude mice (n=6/group). Six weeks post injection, the mice were killed and the lungs were removed and paraffin embedded. Consecutive sections (4μm) were made and stained with hematoxylin-eosin. The micro metastases in the lungs were examined and counted.

### Statistical analysis

All statistical analyses were performed with Statistical Product and Service Solutions (SPSS) 17.0 software (SPSS, Chicago, IL) or Graph Pad Prism 5.0 (Graph Pad Software, La Jolla, CA) software. Data are represented as mean ± SEM. Student’s t-test or one-way analysis of variance (ANOVA) was used for comparisons between groups according to actual conditions. Correlation between expression of NKILA and clinical pathological parameters was analyzed with Fisher’s exact test or chi-square test. Survival curves were generated using the Kaplan-Meier method and assessed using the log-rank test. The Cox proportional hazard regression model was performed to identify independent prognostic factors. Differences were considered statistically significant when *P* < 0.05.

## Supplementary Material

Supplementary File
