# Supplementary material for: NKILA inhibits NF-κB signaling and suppresses tumor metastasis
Source: Aging (Albany NY). 2018 Jan 17;10(1):56–71. doi: 10.18632/aging.101359 (PMC5811242; doi:10.18632/aging.101359)
Supplement: Supplementary File [file aging-10-101359-s001.pdf]

SUPPLEMENTARY MATERIAL

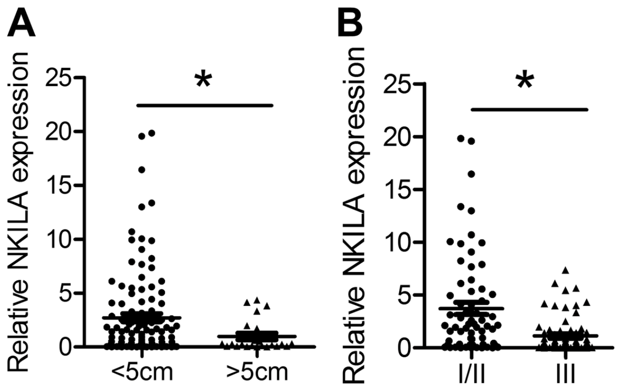

Supplementary Figure 1. NKILA correlates with tumor size and stage. (**A**) Expression level of NKILA in tumor samples with different size. (**B**) Expression level of NKILA in tumor samples of different stage. **P* < 0.05.

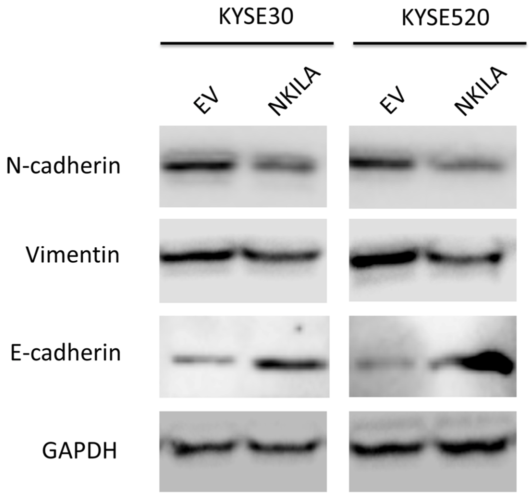

Supplementary Figure 2. Overexpression of NKILA suppresses EMT. Expression level of E-cadherin, N-cadherin and Vimentin in KYSE30 and KYSE520 cells after overexpression of NKILA was detected by western blot.

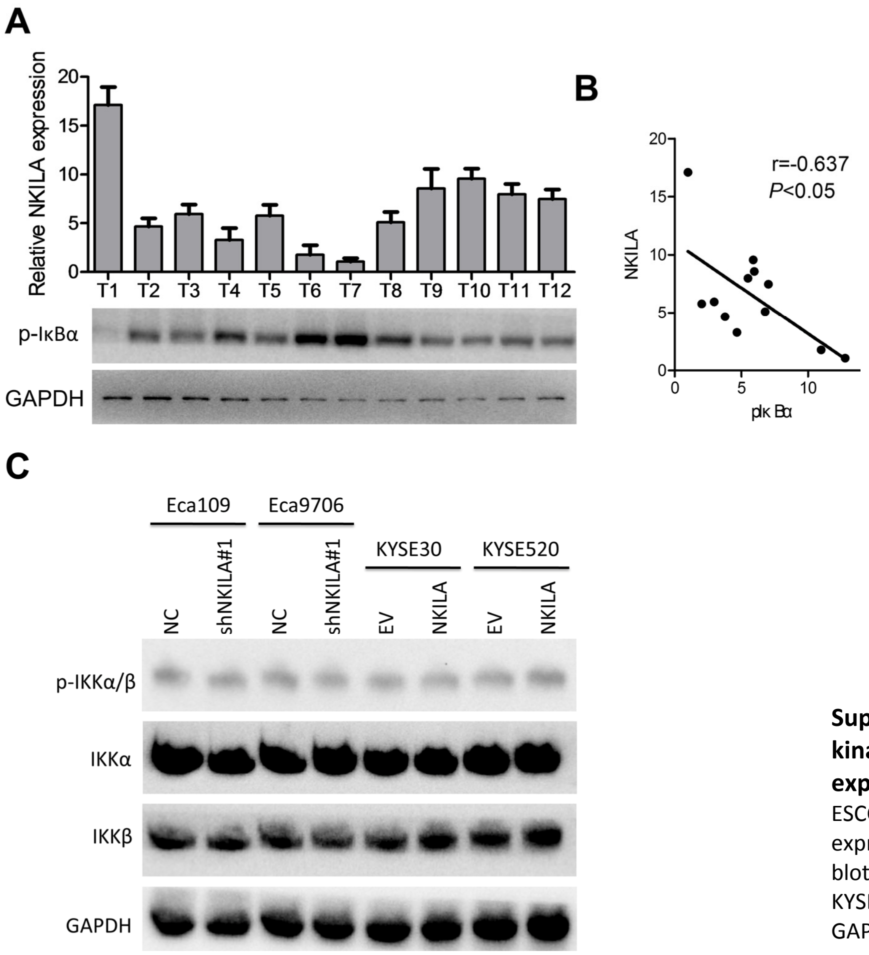

Supplementary Figure 3. Phosphorylation of IκBα kinase (IKK) after manipulation of NKILA expression. (**A**) Expression of p-IκBα and NKILA in twelve ESCC tumor tissues. (**B**) Correlations of p-IκBα and NKILA expression in twelve ESCC tumor tissues. (**C**) Immunoblotting of p-IKKα/β, IKKα and IKKβ in Eca109, Eca9706, KYSE30 and KYSE520 cells after manipulation of NKILA. GAPDH was used as loading control.

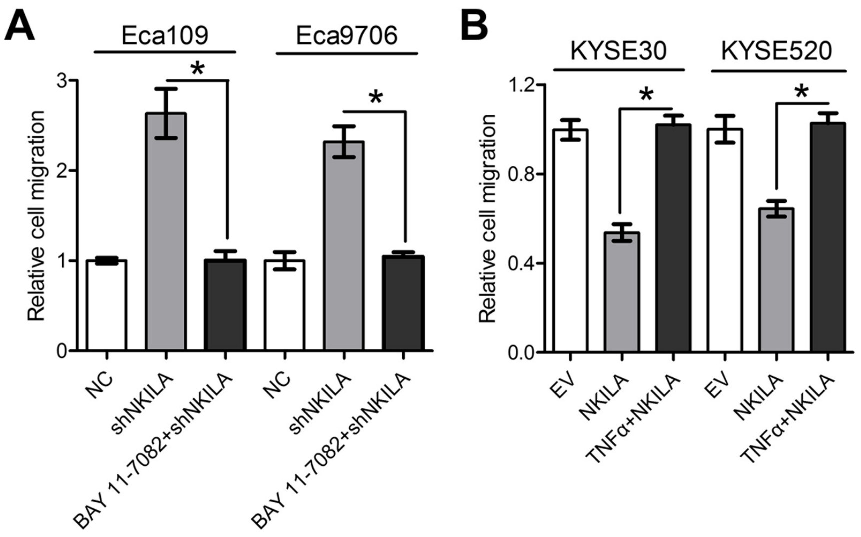

Supplementary Figure 4. NKILA suppresses metastasis of ESCC cells via inhibition of NF-κB signaling. (**A**) Quantification of migration assays after NKILA silencing in Eca109 and Eca9706 cells with pretreatment with BAY 11-7082 (10μM). (**B**) Quantification of migration assays after NKILA overexpression in KYSE30 and KYSE520 cells with pretreatment with TNFα (20ng/ml). Data in A and B represent the mean ± SEM of three repeated experiments. *$P < 0.05$.
